# Resistance Measurements with Dynamometer in Foundation Dorsal Preservation Rhinoplasty Techniques

**DOI:** 10.1007/s00266-025-04834-8

**Published:** 2025-04-09

**Authors:** Veysel Berber, Sercan Gode

**Affiliations:** 1 Department of Otolaryngology, Sarikamis State Hospital, 36100 Kars, Turkey; 2 Department of Otolaryngology, Ege University School of Medicine, Izmir, Turkey

**Keywords:** Preservation rhinoplasty, Dorsal preservation, SPQR, Hump recurrence, Additional maneuvers, Dynamometer

## Abstract

**Introduction and Aim:**

Some recommendations have been reported about nasal hump recurrence and its possible reasons or mechanisms after dorsal preservation rhinoplasty. In this study, we aimed to measure how additional maneuvers affect recoil on the dorsum with the help of a dynamometer during the dorsal preservation rhinoplasty operation.

**Materials and Methods:**

Twelve patients who underwent closed approach dorsal preservation rhinoplasty at the Ege University Hospital ENT department between September and December 2022 were included in this prospective study. During the dorsal preservation rhinoplasty operation in all patients, the pressure values caused by the recoil of the dorsum on the probe of the dynamometer placed on the rhinion were measured and recorded in 4 steps. (1: Push down (PD), 2: Let down (LD), 3: Lateral keystone dissection, 4: Rasping)

**Results:**

Of the 12 patients included in the study, 6 (50%) were female and 6 (50%) were male. The mean age was found to be between 29.9 ± 6.5 (minimum 22-maximum 42). Mean follow-up time was 36.6 ±15.3 (minimum 15 -maximum 60). 3 patients (25%) had a straight nose, 7 patients (58.3%) had a tension nose and 2 patients (16.7%) had a kyphotic nose. The scores of all four stages measured were statistically significant (*p *< 0.001). In the comparison of the stages against each other, there were significant differences between PD-LD, LD-LKA dissection, and LKA dissectionrasping (*p *< 0.001). The scores of all four stages measured were statistically significant (*p*<0.001). In the comparison of the stages against each other, there were significant differences between PD-LD, LD-LKA dissection, and LKA dissection-rasping (*p *< 0.001). In the comparative analysis of nose types with each other, the difference between straight nose and kyphotic nose was found to be significant (*p *< 0.05). There was no significant difference between tension nose and other nose types (*p *> 0.05). There were differences in pressure values according to nose types (Straight, Tension, Kyphotic) and all nose types were found to be statistically significantly affected by additional maneuvers.

**Conclusion:**

The effect of maneuvers performed on areas defined as resistance points in dorsal preservastion rhinoplasty have been evaluated with objective measurement methods. In selected cases, noses with severely angled and kyphotic humps require additional maneuvers, including resection of the bony cap, to flatten the nasal dorsum and the cartilaginous roof.

**Level of Evidence III:**

This journal requires that authors assign a level of evidence to each article. For a full description of these Evidence-Based Medicine ratings, please refer to the Table of Contents or the online Instructions to Authors www.springer.com/00266.

## Introduction

Preservation rhinoplasty, requiring advanced surgical techniques, strategically removes small amounts of bone and cartilage from under the nasal dorsum, reshaping the nose without altering the esthetic lines of the dorsum [1]. However, surgical interventions targeting the components forming the dorsum without directly intervening in the dorsum itself may sometimes be insufficient and lead to suboptimal outcomes. The most prominent of these is the recurrence of a dorsal hump in the long term after dorsal preservation rhinoplasty [2–4]. Various modifications have been described in the literature to overcome this situation.

Dorsal preservation is divided into foundation techniques and surface techniques [5]. In surface techniques, hump is treated superficially by modulation of the middle vault without impaction osteotomies, while foundation techniques can be performed with excision of high, middle or low septal strips to correct the cartilaginous convexity of the dorsum and osteotomies necessary for mobilization of the nasal pyramid. The “Simplified Preservation Quick Rhinoplasty” (SPQR) described by Finocchi is a subgroup of foundation techniques based on low septal strip removal as a modification of the Cottle septoplasty [6].

Surgeons performing dorsal preservation (DP) may encounter complications such as saddle nose, asymmetry of the bony roof, cerebrospinal fluid (CSF) leakage, radix step-off deformities or hump recurrence. Among these complications, recurrence of the dorsal hump is one of the most common reasons patients revisit their doctor. This recurrence is rooted in the resistance points formed by the components constituting the bony roof of the nose. Insufficiencies in one or more of these resistance points can lead to a rebound effect on the dorsum post-operation, causing hump recurrence.

Some maneuvers and additional interventions targeting these resistance points have been described by surgeons who perform foundation dorsal preservation rhinoplasty techniques in their current practice. There are no numerical data on how much these additional interventions affect the resistance points. Studies demonstrating the measurability of the rebound caused by resistance using a dynamometer exist. By preparing a mechanism that keeps the dynamometer fixed at the zero point of the rhinion, the instantaneous contact of the recoil force with the probe after the septum that is pulled forward is released can provide quantitative information. This study aims to measure, using a dynamometer, how additional interventions during preservation rhinoplasty SPQR operation affect the recoil of the dorsum.

## Material - Method

**Fig. 1:**
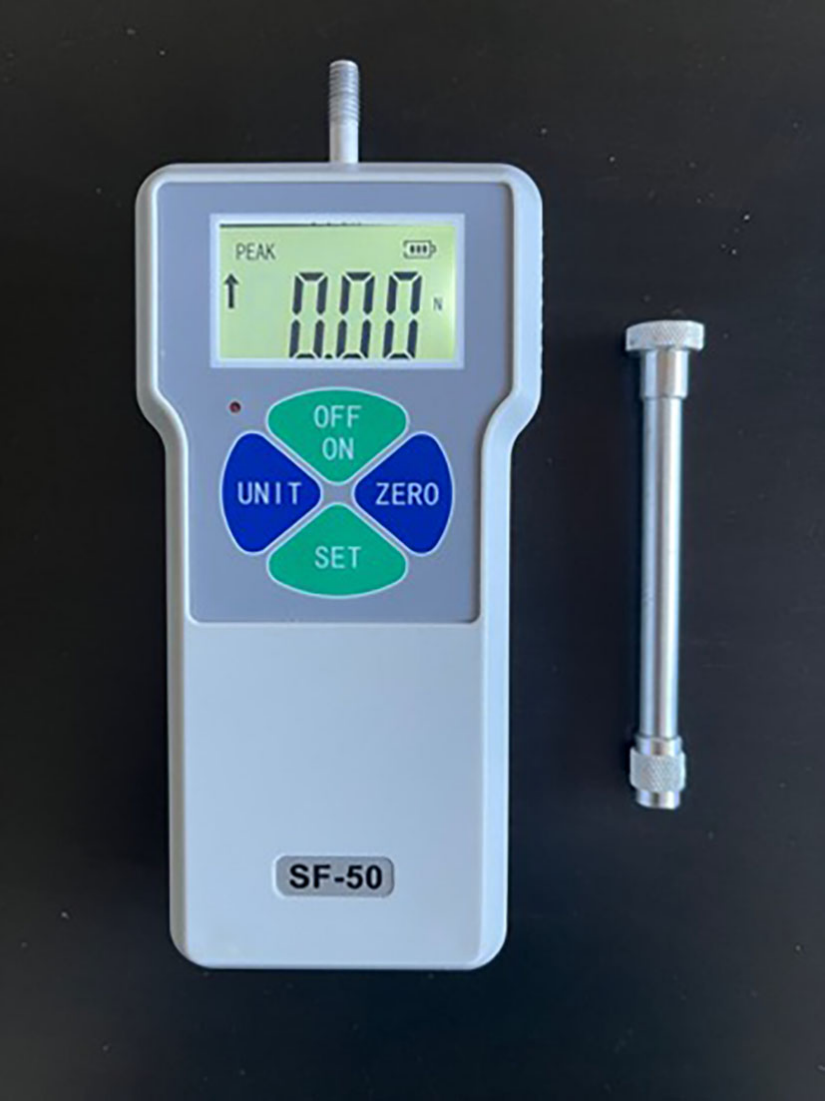
Digital dynamometer

This prospective study included 12 patients who were operated on at the Department of Ear, Nose and Throat, Ege University Faculty of Medicine Hospital, between September 2022 and December 2022. Patients were categorized according to the nose types (straight, tension, kyphotic) classification defined by Saban [7]. All patients underwent subgroup of foundation dorsal preservation rhinoplasty called “Simplified Preservation Quick Rhinoplasty” (SPQR) by the same surgeon (SG). Immediately after the 4 steps of the operation: (1) pushdown, (2) letdown, (3) LKA dissection and (4) rasping, all measurements were taken with an Ege Rate SF-50 (Ege Rate Elektronik, İstanbul) digital dynamometer and numerical data were noted (Fig. 1).

### Surgical Technique

**Fig. 2:**
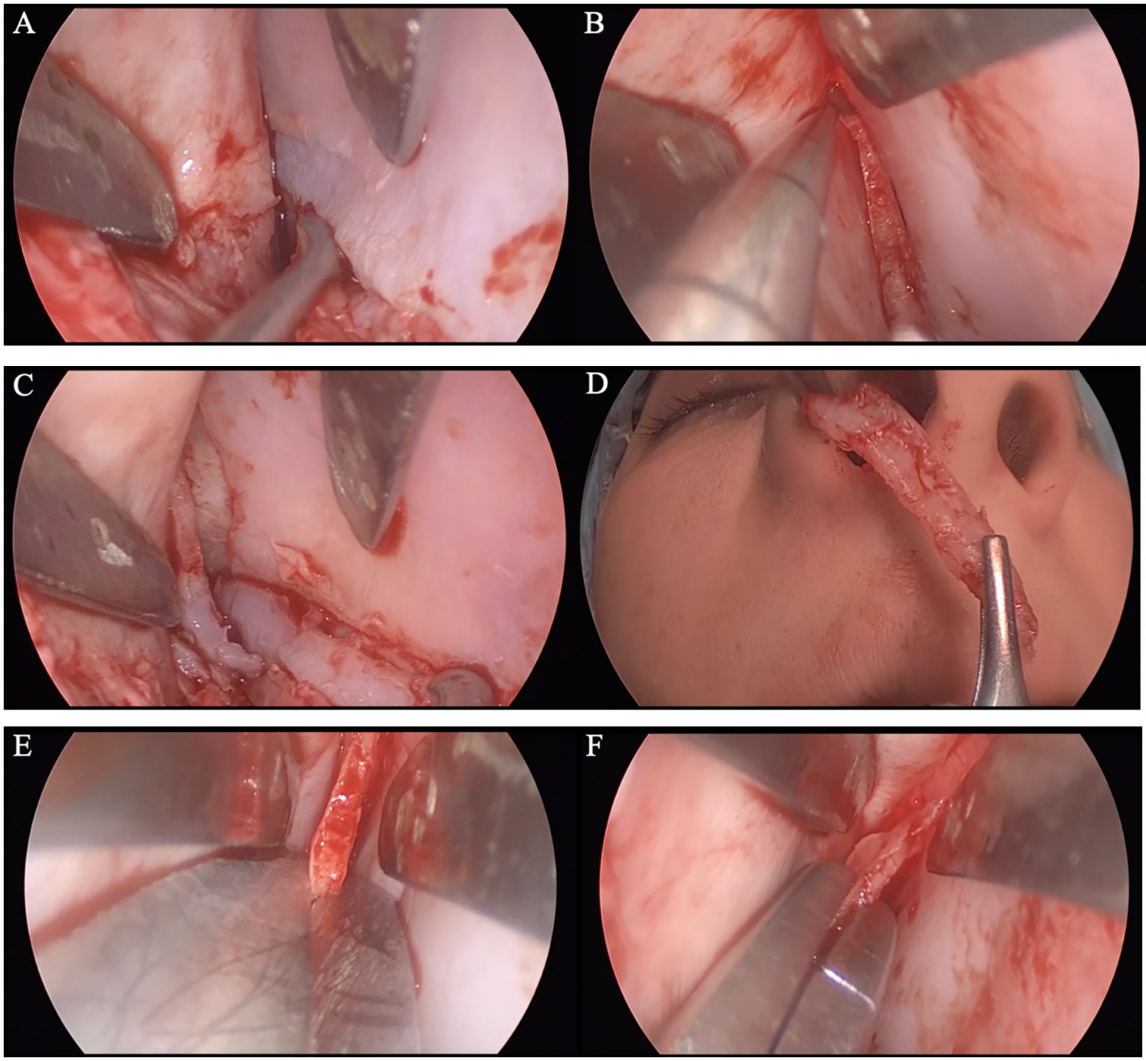
SPQR operation steps. **A** Separation of the septum from the maxillary bone up to the vomer. **B** Vertical incision from the vomer to the rhinion. **C**, **D** Excision cartilage strip from the septum base. **E**, **F** Subdorsal PPE incision and triangular bony piece excision

**Fig. 3:**
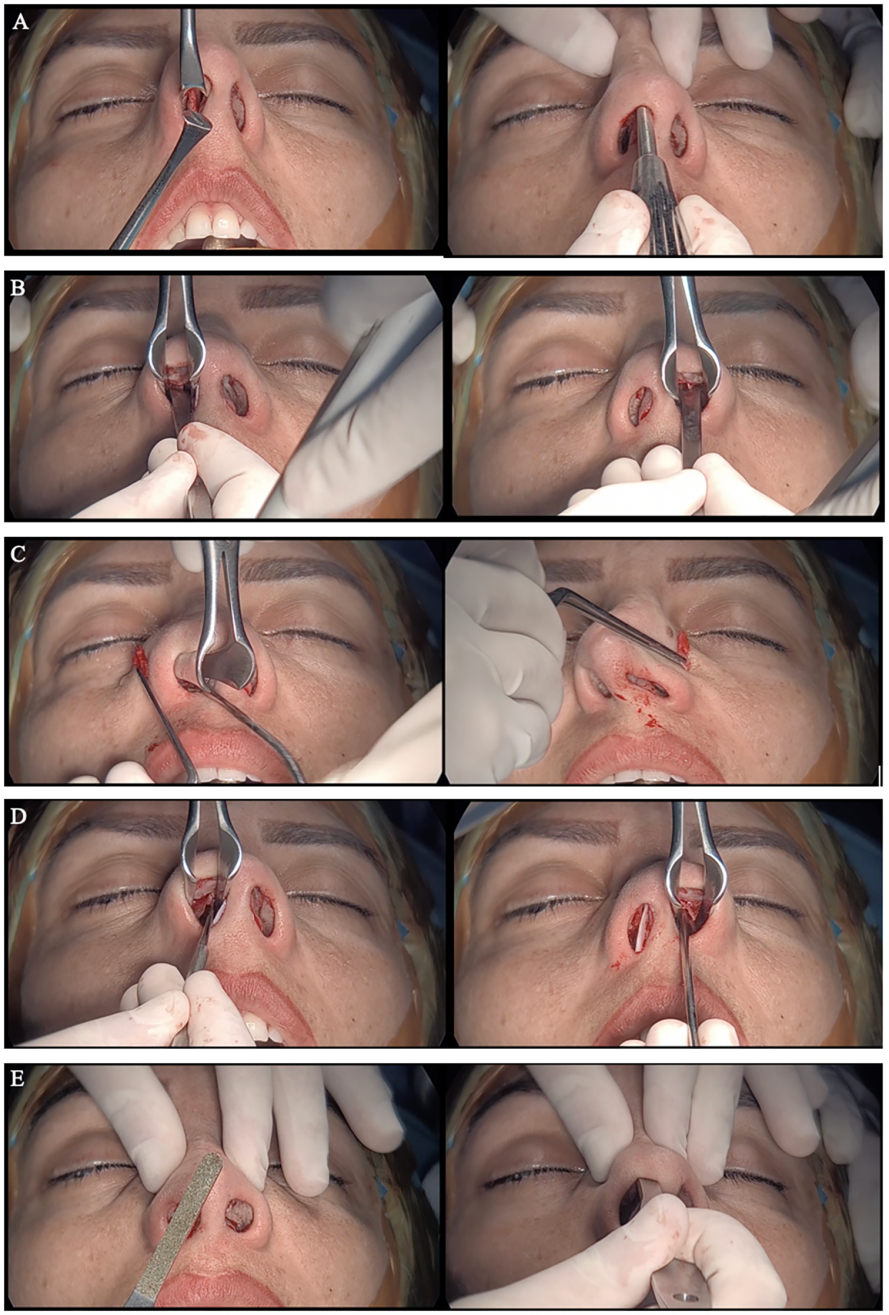
Measurement stages: **A** Transverse osteotomy. **B** Lateral osteotomies. **C** Wedge resections from the piriform aperture. **D** LKA dissections. **E** Dorsum rasping

**Fig. 4:**
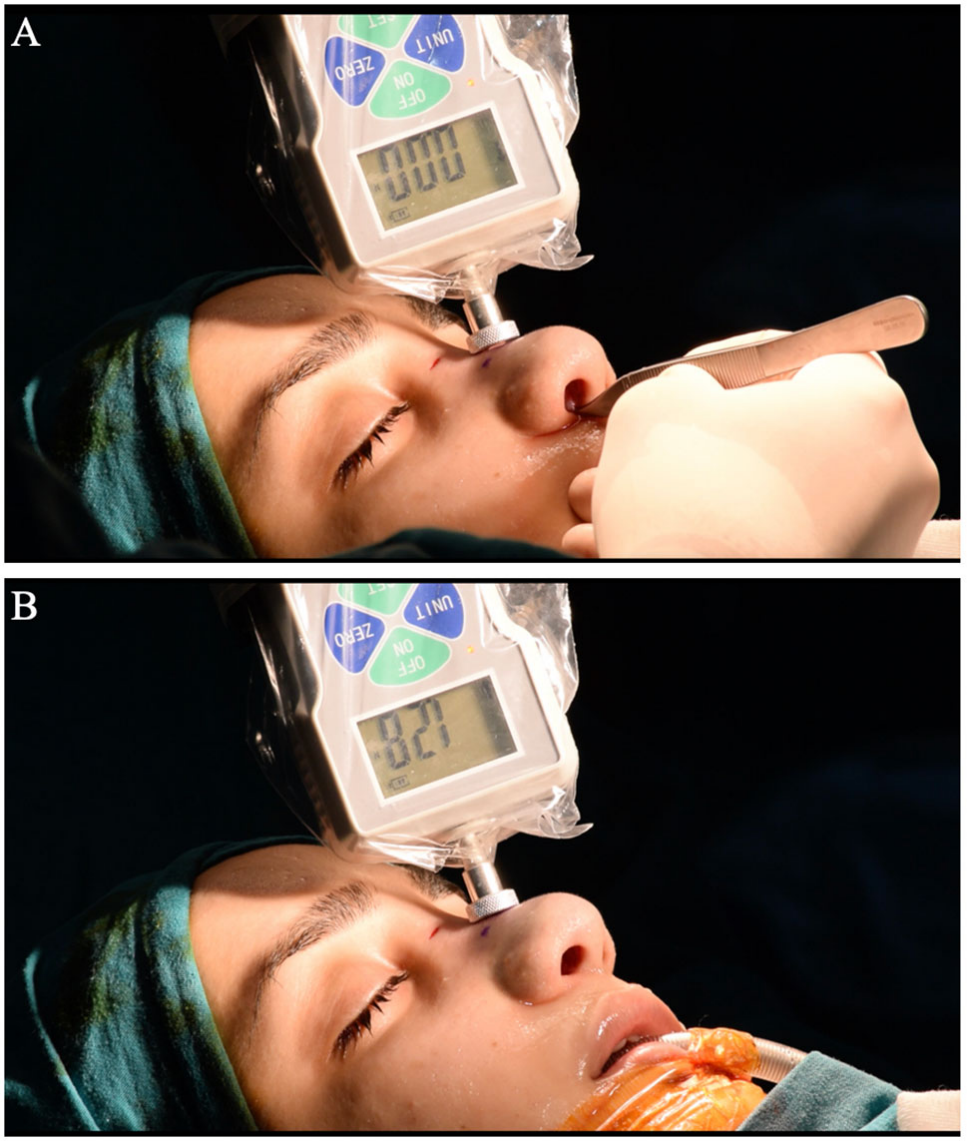
Measurement of recoil pressure from the rhinion with a dynamometer. **A** While the dynamometer is at zero point on the skin in the rhinion, the septum is pulled forward with a rotation movement, allowing the dorsum to flatten. **B** After the septum is released, measurement of the recoil of the dorsum in the rhinion

All patients underwent SPQR with a closed technique approach. After transfixation and bilateral marginal incisions, the septum was dissected in the subperichondrial plane and the septal mucosal flaps were elevated. The nasal dorsum, and upper and lower lateral cartilages were dissected on the supraperichondrial subsmas and the nasal bone in the subperiosteal plane up to the glabella. A “swinging-door” septoplasty was performed by releasing the quadrangular cartilage—anterior nasal spine (ANS), premaxilla, vomer and perpendicular plate of the ethmoid bone. Resection was performed by cutting a strip from the base of the septal cartilage close to the maxillary spine. A vertical incision was made from the vomer to the highest point of the nasal dorsum (usually the rhinion). The subdorsal 2–3 mm triangular bone was excised with a cutting forceps. In order to mobilize the nasal pyramid, first a transverse osteotomy was performed. Then, an incision was made from the piriform aperture and a lateral low-to-low osteotomy was performed with a 3 mm osteotome up to the medial canthus level and connected with the transverse osteotomy line. At this stage, the septum was pulled forward with the help of forceps and the dynamometer was placed at the zero point in the rhinion region. Then, the septum was released and the pressure value created by the recoil of the dorsum on the dynamometer was noted in peak mode in newtons (n). Then, bone wedge was resected with from the piriform aperture at the lower level of the lateral osteotomies to the level of the transverse osteotomy. Again, a second low-to-low lateral osteotomy was performed below the previous osteotomy line with a 3 mm osteotome, and a triangular bone wedge was removed at the level of the nasofacial groove. Thus, the letdown stage was completed. At this stage, the second measurement was taken with a dynamometer. In order to further reduce the recoil in the dorsum, the osteocartilaginous connection between the upper lateral cartilage and the nasal bone was weakened by dissecting the lateral keystone area (LKA) and the third measurement was taken with a dynamometer after this maneuver. Finally, rasping was made to weaken the connection between the nasal bone and the upper lateral cartilages in the dorsum, and finally, the fourth measurement was made with a dynamometer. Then, the caudal end of the septum was fixed to the maxilla anterior nasal spine periosteum with one 5/0 polydioxinone (PDS) and one 5/0 prolene suture. After the part of the operation related to the dorsum included in the study was completed, tipplasty was performed and the operation was completed (Figs. 2, 3, 4).

### Statistical Analysis

Statistical analyses were performed using IBM SPSS Statistics for Macintosh, version 29.0 (IBM Corp. Released 2022. Armonk, NY: IBM Corp), software. Descriptive data are given as Mean ± Standard Deviation. The Shapiro–Wilk test was used for determining the distribution pattern of the data. Differences between consecutive measurements were analyzed using the “repeated measures ANOVA” test, and the Bonferroni test was used as a multiple comparison test to determine the stage at which differences occurred. The *p* < 0.05 value was considered statistically significant.

## Results

The mean age of the 12 patients, 6 (50%) females and 6 (50%) males, was between 29.9  ± 6.5 years (min 22–max 42). The mean follow-up period was 36.6 ±15.3 years (min 15–max 60). According to the classification of nose types defined by Saban, there were 3 patients (25%) with a straight nose, 7 patients (58.3%) with a tension nose and 2 patients (16.7%) with a kyphotic nose. The distribution of age, gender, follow-up period and nose types is shown in Table 1.

**Table 1:** Distribution of age, gender, follow-up period and nose types

	Age	Gender	Follow up (day)	Nose type
1	22	F	60	Tension
2	42	F	58	Kyphotic
3	26	F	55	Straight
4	41	M	48	Tension
5	26	F	39	Tension
6	33	M	35	Straight
7	28	M	32	Tension
8	25	F	28	Kyphotic
9	30	M	26	Tension
10	23	M	25	Tension
11	34	F	19	Tension
12	29	M	15	Straight

All scores measured in a total of 4 stages (1: PD, 2: LD, 3: LKA dissection, 4: Rasping) during the operation were in normal distribution. (*p *> 0.05) The pressure values reflected on the dynamometer at each stage for all patients are shown in Table 2.

**Table 2:** Pressure values of the measured stages

	PD (N)	LD (N)	LKA dis. (N)	Rasping (N)
1	1.28	0.9	0.74	0.48
2	1.8	1.32	1	0.79
3	1.35	1.18	0.52	0
4	1.82	1.41	0.88	0.44
5	1.42	1.24	0.98	0.62
6	1.6	1.32	0.65	0.3
7	1.42	1.07	0.74	0.46
8	1.98	1.38	1.07	0.77
9	1.63	1.34	1.03	0.56
10	1.14	0.85	0.54	0
11	1.48	1.21	0.84	0.53
12	0.97	0.66	0	0
Mean ± Std dev	1.49 ± 0.29	1.15 ± 0.23	0.74 ± 0.3	0.41 ± 0.28

*PD*: Pushdown, *LD*: letdown, *LKA dis* lateral keystone area dissection

In repeated measurements, the scores of all four stages applied in the ANOVA test were found to be statistically significant. (p<0.001) In the comparison of the stages against each other, the differences between PD–LD, LD–LKA dissection and LKA dissection–rasping were found to be significant in the Bonferroni multiple comparison test (*p *< 0.001) (Table 3).

**Table 3:** Comparative statistical analysis of measurement stages with each other

	Stage	Mean(N)	Std. Dev	*P*
1	2	0.33	0.035	<0.001
	3	0.74	0.052	<0.001
	4	1.08	0.058	<0.001
2	1	−0.33	0.035	<0.001
	3	0.41	0.051	<0.001
	4	0.74	0.064	<0.001
3	1	−0.74	0.052	<0.001
	2	−0.41	0.051	<0.001
	4	0.34	0.043	<0.001
4	1	−1.08	0.058	<0.001
	2	−0.74	0.064	<0.001
	3	−0.34	0.043	<0.001

In the comparative analysis of nose types with each other, the difference between the straight nose type and the kyphotic nose type was found to be significant (*p *< 0.05), while no significant difference was found between the tension nose type and the other two nose types (*p *> 0.05) (Table 4).

**Table 4:** Comparative statistical analysis of nose types with each other

	Nose type	Mean (N)	Std. Dev	*P*
Straight	Tension	− 0.25	0.14	0.300
	Kyphotic	− 0.55	0.18	0.044
Tension	Straight	0.25	0.14	0.300
	Kyphotic	− 0.30	0.16	0.291
Kyphotic	Straight	0.55	0.18	0.044
	Tension	0.30	0.16	0.291

**Fig. 5:**
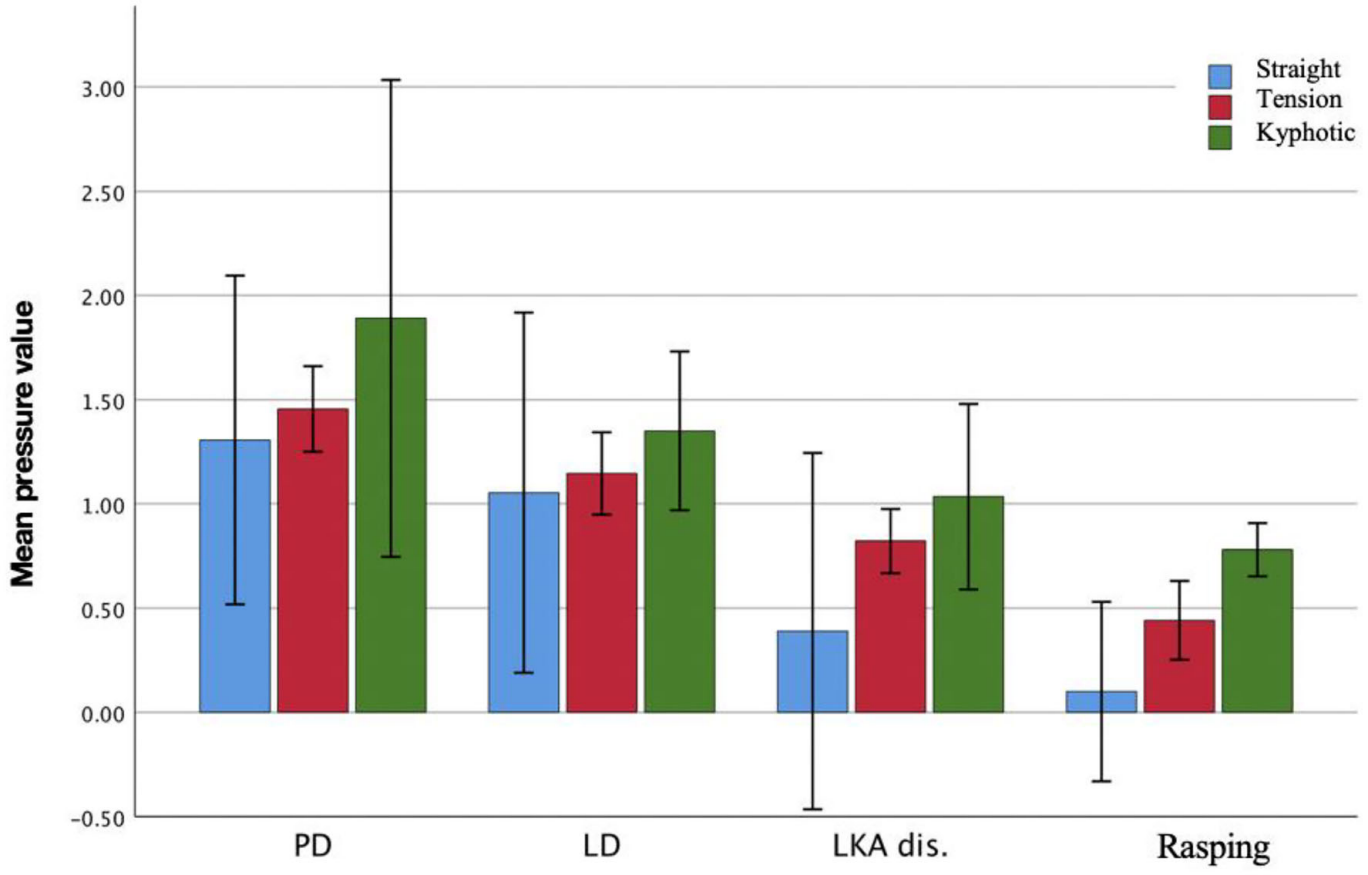
Change graph of mean pressure values in measurement stages according to nose types. PD: Pushdown, LD: letdown, LKA dis: lateral keystone area dissection

There were differences in pressure values according to nose types (Straight, Tense, Kyphotic) and all nose types were found to be significantly affected by additional maneuvers. There was a significant decrease in pressure values in nose types according to the maneuvers applied, respectively (Fig. 5).

## Discussion

Early postoperative period humps can be defined as residue rather than recurrence, because of the difficulty of performing dorsal preservation methods during the learning curve and relatively conservative approaches. In foundation dorsal preservation techniques, there are resistance points that prevent the dorsum from being brought to the desired new position. The spring effect refers to the tendency of the bony–cartilaginous convexity to reappear either intraoperatively or postoperatively after dorsum preservation techniques. The septum and upper lateral cartilages have a natural elasticity that allows them to return to their original shape when repositioned. Even if the bony framework is properly repositioned, soft tissue memory or tension within the perichondrium and periosteum can contribute to dorsal irregularities or minor dorsal elevation over time. By removing the septal strip, the structural resistance created by the septum is broken. It is important to extend the strip excision under the bony dorsum to the radix osteotomy. In most cases, this is cartilage and can be easily resected. If there is bone instead, it should be removed with a rongeur, piezo or osteotome. One of the key concepts of dorsal preservation is to stretch and flatten the released septal cartilage by pulling it posteriorly and caudally. Toriumi reported that he successfully applied this manipulation in Finocchi's SPQR, Neves' Tetris and subdorsal Z flap techniques [8]. Ishida et al. reported a partial hump recurrence of 15% in 120 patients who underwent preservation rhinoplasty with preservation of the cartilaginous roof, as a result of the difficulty in determining and calculating the size of the septal strip to be resected and also the memory of the soft tissues [2]. Saban et al., who advocated high septal strip excision, reported the lowest hump recurrence rate in the literature as 3.4%, and based this on 2 principles: (1) Septal strip excision should be performed just below the dorsum, leaving no residue and (2) reliable fixation of the septum with the upper lateral cartilages [4]. Saban, in another study on the selection of the appropriate technique for the patient for preservation rhinoplasty, found the overall revision rate to be 9.94% and stated that hump recurrence is associated with failure to follow the recommended surgical steps and/or selection of a procedure that is not appropriate for the morphology or expectations of a particular patient. We used the classification he made according to nose type in this study and examined the effects of the same maneuvers on different nose types [7]. Atolini and colleagues reported similar results in 153 patients who underwent the SPAR method, and reported that 13 patients required revision due to hump recurrence [9]. Kovacevic reported a revision rate of less than 10% in 205 patients who underwent the subdorsal triangle resection method [10]. In light of this information, in most studies, patients with favorable anatomical characteristics were selected for specific techniques, which may have influenced the reported recurrence rates. This selection bias could lead to an underestimation of the recurrence risk in a broader patient population. The experience of the senior surgeon (SG) in the SPQR procedure before this study draws attention to three basic steps that may cause hump recurrence. The first is inadequate low strip excision, the second is the failure to make the septal vertical incision completely below the dorsum, and as a result, the resistance of the hinge region cannot be broken sufficiently, and the third is the failure to properly fix the caudal septum to the anterior nasal spine. However, it has been observed that even if these steps are successfully applied, hump recurrences may occur over time. In light of this information, two headings emerge to prevent hump recurrences. The first is complete mobilization of the nasal pyramid after the necessary septal excision, and the second is fixation of the components with sutures. In our study, a similar surgical procedure was performed by the same surgeon for all patients. In this context, first of all, a low septal strip excision was performed in order to reduce the dorsum sufficiently. Thus, the septal resistance force was overcome. After the median and lateral osteotomies were completed, the nasal pyramid was completely mobilized.

Webster triangle is another point that will create resistance during the shaping of the dorsum. In PD, the osteotomy line weakens this point. In LD, excision of the bone wedge breaks the resistance in Webster triangle in the caudal part. It has been stated that LD osteotomy can provide more effect than PD osteotomy in cases where the dorsum needs to be lowered more than 4 mm [4, 11]. In the PD technique, the nasal base should be limited to avoid internal nasal valve obstruction by shifting the nasal bones inside the piriform aperture with a narrowing effect, whereas the LD technique involves a bone wedge resection from the maxilla. Thus, the bone fragments come into contact with each other and it is possible to lower the roof without any base narrowing. Tuncel and Aydoğdu reported a rate of 12% in their study on the causes of hump recurrence after LD and PD [12]. In this study, it was determined that recurrences were more common in patients with humps greater than 4 mm, and this rate was reduced to 5.3% with maneuvers such as vertical incisions just below the key area, LKA dissection and preference for the LD procedure in kyphotic noses, and appropriate patient selection. They stated that all of the hump recurrences after these additional maneuvers occurred in patients in whom the dorsum had to be lowered more than 4 mm [13]. In our study, it was determined that the recoil pressure in the nasal dorsum decreased more after LD compared to after PD. Although this decrease was greater in kyphotic nose types, a statistically significant result could not be reached due to the small number of patients. Although it has been stated that the superiority of LD over PD occurs in cases where the dorsum needs to be lowered more than 4 mm, the majority of the patients included in the study were those for whom lowering the dorsum less than 4 mm would be sufficient. The data support that excision in the Webster triangle can reduce recoil in the nasal dorsum for all nose types.

Especially in tension and kyphotic noses, it is thought that the main problem is not only reshaping the bony cap and making the DKA more flexible, but also releasing the LKA, since the lateral resistance forces are the main problem. Again, studies have focused on two basic components for the convex dorsum, namely the bone and cartilaginous hump, and it has been argued that classical pushdown application without an additional maneuver for dorsums with a pronounced hump will not be sufficient to obtain a flat dorsum, and additional maneuvers such as rasping/reshaping for the bony hump and releasing the LKA for the cartilaginous hump should be applied [5]. Ishida [2] and Jankowski [14] have proposed dorsum lowering via nasal bony–cartilaginous disarticulation as their preferred technique. In the “Balerina maneuver” defined by Göksel [15], it is aimed to completely release the LKA at the junction of the upper lateral cartilage and the nasal bone without damaging the inner mucosa and to weaken the lateral resistance point. Since the dorsal height of the nose is not a two-dimensional structure, it is argued that in order to change the shape of the dorsum, in addition to the release in the dorsal key area (DKA) region, the lateral key area (LKA) area should also be moved. Without this dissection, the shape of the dorsum would have descended without changing, which does not comply with one of the basic goals of flattening the dorsum. As a result, with this maneuver, the upper lateral cartilage is released and the lateral wall connections are mobilized without tension, allowing a cartilaginous descent and avoiding the “spring effect” or pop-up phenomenon [16]. Although LKA alone does not cause the spring effect, the lateral side walls and scroll ligament connections also contribute to it. Toriumi argues that he does not prefer LKA dissection in dorsums with relatively small humps, and that it is important and necessary to apply it especially in patients with larger dorsal humps [8]. In our study, it was found that recoil pressure decreased significantly in all nose types after LKA dissection. We think that there is an important resistance point that needs to be overcome in tension and kyphotic nasal structures. The data obtained indicate that LKA release can be effective even in straight nasal structures. Since LKA dissection is applied to all patients, whether they have a straight, tension or kyphotic nasal structure, it is not possible to say which dorsum height should be preferred in this sense. This seems to be a situation that can only be evaluated after long-term follow-up of groups that include patients with similar nasal structures and that do and do not undergo LKA dissection.

The length of the bony roof is a critical determinant of how easy it will be to flatten the hump. Flattening long nasal bones is more difficult because cartilage is more flexible than bone. Saban stated that in some patients with postoperative hump recurrence, simple rasping can be performed without opening the roof [4], and he made some recommendations for hump residues that occur during surgery. First, he recommended that subdorsal septal remnants be checked in patients with high septal strips, and if present, they should be excised with forceps or weakened by making vertical incisions. If hump residue persists, he suggested that the bony cap can be rasped with conventional or piezoelectric instruments [17]. Göksel suggested that rasping be performed in order to weaken the connection of the nasal bones to the upper lateral cartilages on the dorsum, in addition to the ballerina maneuver he described [18]. Tuncel and Aydoğdu recommend rasping the nasal bone in cases with kyphotic nasal structure because the nasal bone is both longer and higher [12, 13]. In our study, it was determined that the maneuver to weaken the DKA resistance by rasping the dorsum was significantly effective in all nasal structures. In patients with a flat nasal structure, rasping may not be necessary since the recoil force has decreased considerably up to this stage.

In the current literature, there are articles based on observational results and surgical experience regarding the expansion of the indications for preservation rhinoplasty, its modifications and the definition of additional maneuvers. There is no study based on objective measurement that can evaluate the effectiveness of these additional maneuvers on humans and their superiority over each other. In this study we determined and measured 4 stages that weaken blocking points. These stages alone may not be enough to break all resistance. The natural shape of the upper lateral cartilages, ligaments and their connections, soft tissue formation during the healing process and inadequate fixation may also contribute to hump recurrence.

All measurements were taken by the same surgeon in the “peak” mode with a dynamometer fixed to a cantilevered platform. In this mode, the highest value of the initial distortion in the rhinion after applying pressure was fixed on the device. Since the value is recorded at the first touch, longer and stronger pressure on the probe does not change the result. Repeated measurements were taken to improve reliability. There are three studies in the literature where a similar device was used for similar purposes [19–21]. It was stated in two studies that a fixed value could not be easily determined due to the high sensitivity of this device. Therefore, the measurements were recorded with a camera and later analyzed in a computer environment. However, in an experimental study on L-strut biomechanics on a lamb's head model, it was reported that this problem was solved by using the "peak" mode of the device [21]. Our limitations in this study are the small number of patients, the lack of long-term follow-up of the patients and the difficulty of standardizing the values obtained due to the high sensitivity of the dynamometer device.

## Conclusion

The most important factor for foundation dorsal preservation techniques and its modifications is patient selection. It is important to determine the type of nose and choose the appropriate method for DP indication. Although additional maneuvers have been shown to be effective in eliminating the hump in patients with a flat dorsum, only PD or LD may be sufficient. In noses with a severely angular and S-shaped hump, additional maneuvers, including bone cap resection, are needed to flatten the nasal dorsum and cartilage roof to minimize the risk of revision. Our results support the necessity of additional maneuvers in tension and kyphotic nasal structures. Our study is the first study in the literature to obtain numerical data on the effectiveness of additional maneuvers in humans undergoing subgroup of foundation dorsal preservation rhinoplasty techniques, and clearer data can be obtained with larger patient groups and more standardized measurement methods.
